# Progress in Sterilization Legislation

**Published:** 1913-02

**Authors:** 


### Progress in Sterilization Legislation.

The *Journal of the American Medical Association* summarizes the progress so far made by States in the matter of legislation prohibiting procreation of the unfit by sterilization. Many inquiries have been received recently regarding the progress of State legislation on the sterilization of criminals. In order to collect all the data on this subject in a single article, it seems advisable to review the history of this movement. The first law on this subject was adopted by Indiana in 1907. The law is entitled "An Act to Prevent Procreation of Confirmed Criminals, Idiots, Imbeciles and Rapists," and provides that each public institution of the State entrusted with the care of such persons shall have on its staff, in addition to the regular institutional physician, two skilled surgeons of recognized ability, whose duty it shall be, in conjunction with the chief physician of the institution, to examine the mental and physical condition of such inmates as are recommended by the institutional physician and the board of managers. If, in the judgment of this committee, procreation is inadvisable, and there is no probability of improvement of the mental and physical condition of the inmate, the surgeons are authorized to perform "such operation for the prevention of procreation as shall be safest and most effective."

In 1909, Washington, California and Connecticut passed similar laws. The Connecticut law provides for a board consisting of two skilled surgeons and the physician in charge of the institution. If this board decides that procreation by any inmate reported to them by the warden or superintendent would produce children with an inherited tendency to crime, insanity, feeble-mindedness, idiocy or imbecility, and that there is no probability that the condition of any such person so examined will improve to such an extent as to render procreation by any such person advisable, then the board shall appoint one of its members to perform the operation of vasectomy or oophorectomy on such person. It will be noted that the Connecticut law is more specific in that it specifies the conditions which would render procreation inadvisable, and also specifies the operation to be performed.

In 1911, New Jersey, Nevada and Iowa adopted similar laws. The New Jersey law is somewhat more detailed than those previously passed. It provides for a surgeon and a neurologist appointed for five years, who, in conjunction with the Commissioner of Charities and Correction, shall be known as the Board of Examiners of Feeble-Minded, Epileptics, Criminals and Other Defectives. Those coming under the operations of the law are limited to persons convicted of the crime of rape or of such successions of offenses against the criminal law as in the opinion of the board shall be deemed sufficient evidence of confirmed criminal tendencies. The opinion of the board under the New Jersey law must be unanimous instead of merely a majority, and before performing the operation the board must apply to some judge of the Court of Common Pleas for an order authorizing the operation. The court is authorized to assign counsel to represent the individual under examination, and all such orders are subject to review by the Supreme Court. Surgeons performing the operation under the law are relieved from responsibility. A record is required of each examination, and a report from the superintendent is called for one year after each operation, showing the result of the operation and its effect on the individual.

In 1912, New York adopted a law providing for a board composed of one surgeon, one neurologist and one practitioner of medicine, each with at least ten years' experience, to be appointed by the Governor for five years. The provisions regarding those coming under the operation of the law are similar to those in the New Jersey act. The board of examiners is required to apply to any judge of the Supreme Court (which, in New York, is the court of first and not of last resort), or to the county judge of the county in which the person is confined, asking for the appointment of counsel to represent the person to be examined. The same safeguards are provided as in the New Jersey law, as well as the same provisions regarding reports, etc. An additional clause is added, forbidding the performance of this operation on any person except as authorized by the act, or except as a medical necessity.

A comparison of the laws of Indiana, the first adopted, and New York, the last one, shows that the subject has been considerably amplified since its inception, additional safeguards having been thrown around it. While every possible precaution should be taken to avoid injustice to any individual, it seems doubtful whether the provision of the New Jersey law, permitting the appeal in each case to the highest court of the State, is advisable. The provision requiring an order of the court based on facts presented by the board in open court, in which the individual under consideration is given the right of appearance by counsel, would seem to afford ample safeguard against injustice. Unlimited right of appeal in each case to the highest court of the State hardly seems justifiable, as this provision could easily make it possible to block the operation of the law. The subject is still in a formative condition.

Eight States have so far taken action. It is probable that a number of others will consider the subject seriously during the coming winter. The Supreme Court of Washington is the only court of last resort to pass on the question so far. On September 3 the court handed down a decision sustaining the law and affirming its constitutionality. The court held that the operation was neither painful nor dangerous, and that punishment which included such an operation was not cruel or inhuman. As the principal provisions of the Washington law are found in all those enacted, it is probable that this decision will be accepted as establishing the right of the State to render procreation impossible as a punishment for certain crimes. The right of the State to impose such treatment on a defective ward of the State has not yet been reviewed by the courts.

[Similar bills are now before the Legislatures of Texas and Minnesota. In Minnesota in the twenty years from 1884 to 1904 (census report) the population increased 59.7 per cent; the feeble-minded increased 380.3 per cent. What will the progeny be, of this horde of feeble-minded.—Ed.]

---

The following is a simple and usually satisfactory operation for ingrown toenail that has progressed beyond palliative treatment: Beginning at the free margin of the nail about a quarter of an inch from the offending side, with straight, strong, narrow-bladed, probe-pointed scissors cut through the length of the nail and continue *under the skin*, directly through the root. With forceps loosen and lift out the narrow segment of nail and nail root complete. Be sure no fragments remain. The operation is brief and the pain, even if no anesthetic is used, is not very severe. Lightly pack the narrow wound. If there is much infection, apply a wet dressing; otherwise a simple pledget of gauze fastened with adhesive strips. The patient can at once walk with comfort in his street shoes, and the after-treatment is trifling.—*American Journal of Surgery.*

